# The microbiome of the upper respiratory tract in health and disease

**DOI:** 10.1186/s12915-019-0703-z

**Published:** 2019-11-07

**Authors:** Christina Kumpitsch, Kaisa Koskinen, Veronika Schöpf, Christine Moissl-Eichinger

**Affiliations:** 1 Diagnostic and Research Institute of Hygiene, Microbiology and Environmental Medicine, Medical University of Graz, Neue Stiftingtalstraße 6, 8010 Graz, Austria; 2 Institute of Psychology, University of Graz, Universitaetsplatz 2, 8010 Graz, Austria; 3 BioTechMed-Graz, Mozartgasse 12/II, 8010 Graz, Austria; 4 Present address: Medical University Vienna, Spitalgasse 23, 1090 Vienna, Austria

**Keywords:** Microbiome, Upper respiratory tract, URT, Human microbiome, Nasal microbiome, Upper respiratory tract diseases

## Abstract

The human upper respiratory tract (URT) offers a variety of niches for microbial colonization. Local microbial communities are shaped by the different characteristics of the specific location within the URT, but also by the interaction with both external and intrinsic factors, such as ageing, diseases, immune responses, olfactory function, and lifestyle habits such as smoking. We summarize here the current knowledge about the URT microbiome in health and disease, discuss methodological issues, and consider the potential of the nasal microbiome to be used for medical diagnostics and as a target for therapy.

## Introduction

The human microbiome is a complex community of microorganisms, living in a symbiotic relationship in human microhabitats. Due to microbial niche specificity, microbial composition and function vary according to the different human body sites, such as the gastrointestinal tract, skin, and airways [1, 2].

Since a healthy adult breathes more than 7000 l of air a day, the upper respiratory tract (URT) is constantly bathed in airflow from the external environment. Along with the air, 10^4^–10^6^ bacterial cells per cubic meter of air are inhaled per day. Besides these biological particulates, the URT is exposed to atmospheric physical and chemical parameters, including varying humidity, oxygen, immunological factors, or nutrients. Along with the anatomy, these factors shape specific microenvironments in the URT such as the nasal cavity, sinuses, nasopharynx, and oropharynx [3–5]. As a consequence, specific microenvironments in the URT harbor different microbial communities composed of variable proportions of resident and transient microorganisms [6].

Like other human body sites, the upper respiratory tract is colonized by a variety of different microbial species directly after birth. It has been shown that the initial colonization depends on delivery mode (vaginal delivery or caesarean section), and the most drastic changes occur during the first year of life, probably driven by the maturation of the immune system [7]. Later on, this first microbial community transforms into the adult URT microbiome, becoming less dense and more diverse. In the elderly, the distinct microbiomes of specific microenvironments become more similar [8, 9].

Many studies report that the nasal microbiome of healthy humans is primarily composed of the phyla Actinobacteria, Bacteroidetes, Firmicutes, and Proteobacteria with representatives of genera *Bifidobacterium*, *Corynebacterium*, *Staphylococcus*, *Streptococcus*, *Dolosigranulum*, and *Moraxella* predominating [9–12]. However, most research focuses on the bacteria in the human nasal cavity, while other components of the microbiome, such as viruses, archaea, and fungi, are seldom specifically addressed and thus likely overlooked [13].

Human health has been described as the outcome of the complex interaction between the microbiome and its human host [14]. Functional or compositional perturbations of the microbiome can occur at different body sites and this dysbiosis has been linked with various diseases; for example, inflammatory bowel disease and metabolic disorders have been linked to dysbiosis in the microbiome of the gastrointestinal tract and URT infections (URTI, such as chronic rhinosinusitis [CRS]) with dysbiosis in the URT [15–18]. These dysbioses are often characterized by a loss of beneficial, commensal bacteria, which protect against overgrowth of opportunistic pathogenic bacteria [6, 19, 20].

Currently, several different therapies are suggested for the treatment of inflammatory URTIs [21–24]. Antibiotics as well as intranasal corticosteroids are used, combining antimicrobial and anti-inflammatory properties [21, 24]. These treatments cause a loss of microbial diversity, potentially leading to an increase of Gram-negative bacteria in the nose [25–27].

In the case of chronic rhinosinusitis, sinus surgery (aiming at improving drainage of the mucus), combined with different antibiotics is the most common treatment [22]. Although this type of therapy is highly invasive, its outcomes are usually satisfactory [28]. However, airway diseases might also be prevented and treated with less aggressive therapies such as saline rinses, cleaning the nasal mucosa from inflammatory mediators and other pollutants [23].

Comparative URT microbiome research faces various methodological problems, including choice of sampling techniques (e.g., swabs, nasal rinses, and dry filter papers) and sampling sites. In most cases anterior nares, middle meatus, and nasopharynx are the preferred sites for sampling [9, 11, 12, 29–31], as other areas are not easily accessible. This often results in a discrepancy of research question and study protocol, as, e.g., the middle meatus is sampled instead of the sinuses when chronic rhinosinusitis is studied [29]. However, microbiome dysbiosis often extends to locations beyond the sites of the studied disease, so that significant alterations in the microbial community structure in adjacent locations can be observed as well [6, 32]. Nevertheless, in order to prove or reject a research hypothesis, the sampling sites for microbiome analyses need to be chosen wisely [6].

The aim of this review is to summarize the current information about the microbiome in the upper respiratory tract; discuss methodological issues such as sampling methods and sites; present the link between URT microbiome composition, immune system, and certain diseases; have a look at the influence of common therapies on the URT microbiome; and identify the current gaps in our knowledge.

Details of cited studies, including sampling, sample processing protocol, studied population and sites, and results are summarized in Additional file 1.

## Landscape of the upper respiratory tract

**Fig. 1:**
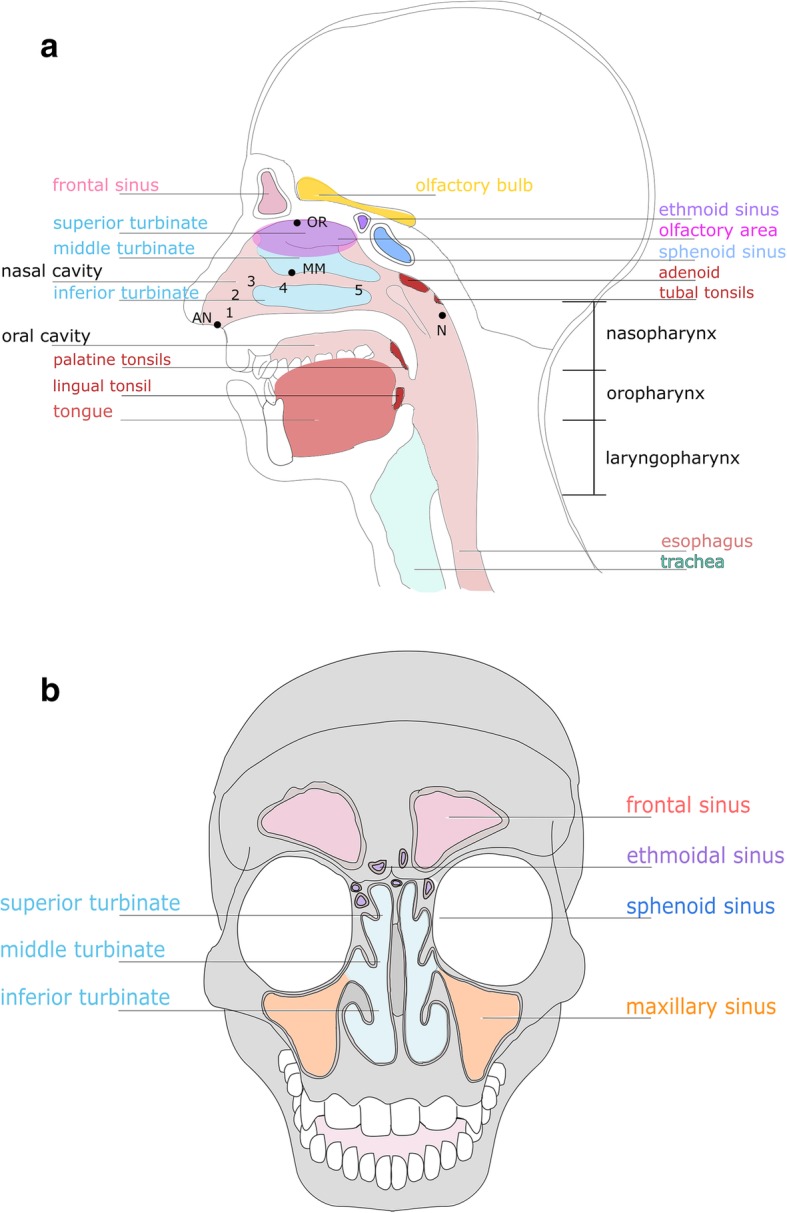
The upper respiratory tract (**a**) and its paranasal sinuses (**b**). **a** URT with different, typical microbiome sampling sites (*AN* anterior naris, *MM* middle meatus, *OR* olfactory area and nasopharynx) and nasal lining, starting with *1* non-keratinized skin-like epithelium in the nostrils followed by different epithelial types, *2* squamous epithelium without microvilli, *3* transitional epithelium with ciliated cells, *4* pseudostratified columnar epithelium with ciliated cells, *5* pseudostratified columnar epithelium with many ciliated cells. **b** Sinuses of the nasal cavity

The upper respiratory tract (URT) comprises the anterior nares, nasal cavity, sinuses, nasopharynx, Eustachian tube, middle ear cavity, oral cavity, oropharynx, and larynx. The nasal cavity is partitioned into the inferior, middle, and superior meatus by three nasal turbinates [3, 33] (Fig. 1a). In this review we focus on the microbiomes of anterior nares, nasal cavity, sinuses, and nasopharynx and their importance in human health.

Many important physiological functions are provided by the URT such as filtering, warming, and humidifying of inhaled air [3, 34]. As the nasal cavity is in constant contact with the external environment, it acts as a physical transition forming an interface between the external environment and the lower respiratory and gastrointestinal tract [3, 33]. Other functions are olfactory sensing and important immunological tasks, including immediate pathogen detection such as sensing of bacterial lactones by taste receptors [32, 35–38].

The nasal cavity is lined by different types of epithelium, providing different micro-niches (Fig. 1a): the anterior naris starts with non-keratinized skin-like epithelium (1), changing into stratified squamous epithelial cells without microvilli (2), followed by transitional epithelium with short microvilli (3), before transition into the middle meatus with its pseudostratified columnar epithelium (4 and 5, middle meatus) [32, 33, 35]. The most common sampling sites for nasal microbiome analyses are the anterior nares (AN), the middle meatus (MM), and the nasopharynx [9, 12, 29, 31] (Fig. 1a).

The surfaces in the *anterior nares* and nasal vestibule are relatively dry compared to other URT areas. These parts experience the greatest exposure to the external environment and contain sebaceous glands (see below) and vibrissae (hair). These hairs trap large particles (> 3 μm) from inhaled air, whereas small particulate matter (0.5–3 μm, including microorganisms) is captured by a flowing mucus blanket covering the entire nasal cavity [32, 33, 35, 39].

The *middle meatus* is adjacent to the nasal vestibule. As it receives drainage from the anterior ethmoids, maxillary, and frontal sinuses, this area is of interest for many microbiome studies [32]. The *nasopharynx* is characterized by many crypts and folds, and its wall is dominated by keratinized and non-keratinized stratified squamous epithelium and pseudostratified ciliated epithelia [40].

**Fig. 2:**
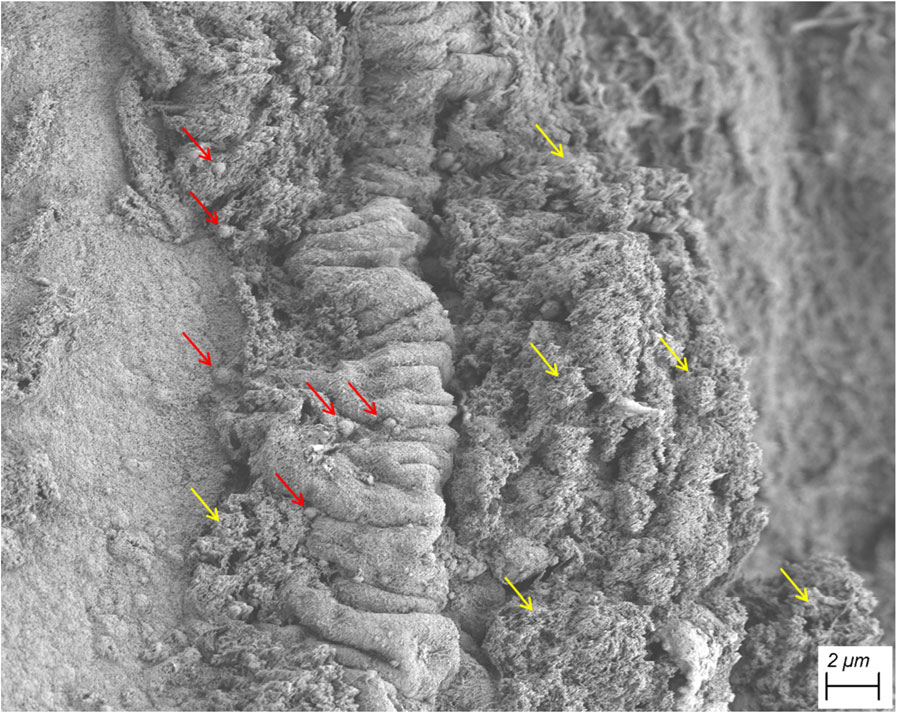
Scanning electron micrograph of nasal mucus of a healthy subject exhibiting various structures (*red arrows* point at bacterial-like structures, *yellow arrows* show areas with nasal phlegm)

Maxillary, ethmoid, sphenoid, and frontal sinuses are air-filled, paired cavities within the facial skeleton, which are important for humidification and warming of the inhaled air (Fig. 1b). They are coated with ciliated columnar epithelium, which produces mucus that is transported into the nasal cavity [41]. These drainages create local micro-niches with specific microbial populations within the nasal cavity [42] (Fig. 2). Another interesting niche for microbiome studies is the *olfactory area*, as recent studies indicated a potential correlation of olfactory function with the taxonomic composition of the local nasal microbiome [43]. The olfactory area is located at the ceiling of the nasal cavity [33].

## Upper respiratory (immune) defense system

The respiratory tract has recourse to a variety of mechanisms, including components of the innate and adaptive immune system, to protect against possibly harmful, inhaled microorganisms while chronically present commensal microbes of the URT microbiome are tolerated due to hyporesponsiveness of the host’s immune system [44].

### The mucus layer

Glands, goblet cells, and ciliated cells secrete a hydrated mucus layer which contains lipids, glycoproteins, and glycoconjugates. This layer not only helps to humidify inhaled air but also traps microbes and microparticles from the environment on entering the URT [33, 45]. This “contaminated” mucus is then directed by ciliated epithelial cells (located in the upper respiratory tract) from the nasal cavity towards the esophagus [33, 45]. This whole process of purging is also known as mucociliary clearance [46, 47]. Additional defense is derived from antimicrobial compounds which are present in the mucus and immediate initiation of immune priming [32, 48]. Interestingly, commensal bacteria with immunomodulatory properties are capable of priming a host’s immune responses to assure efficient and rapid defense against pathogens [49, 50].

### Antimicrobial peptides and reactive oxygen species

The respiratory surface epithelium secretes a variety of antimicrobial components. These include antimicrobial peptides such as lysozyme, lactoferrin or defensins, and reactive oxygen species (ROS) such as hydrogen peroxide and nitric oxide (NO) [51–55]. Besides its antimicrobial activity (it diffuses into the microbial cell and destroys intracellular components), nitric oxide also directly increases mucociliary clearance and speeds up the frequency of ciliary beating by protein kinase G and guanylyl cyclase activation [38, 56–58].

### Nasopharyngeal-associated lymphoid tissue

Nasopharyngeal tonsils (adenoids), the paired tubal tonsils, the paired palatine tonsils, and the lingual tonsil are part of the lymphoid tissue in the nasopharynx and serve as major sites for microbial recognition and defense [59, 60]. Nasopharyngeal-associated lymphoid tissue (NALT) harbors a large variety and number of immune cells, including dendritic cells, macrophages, and lymphocytes [61] (Fig. 1a). Fifty percent of these lymphocytes are immunoglobulin-producing B-lymphocytes [62–64]. Like the small intestine, the lymphoid tissues contain M cells, which transport microorganisms via trans-epithelial transport from the apical surface to the basolateral site where immune cells are already waiting [65]. NALT-associated cells (e.g., sinonasal solitary chemosensory cells) excrete chemokines and cytokines, which activate downstream immune cascades [66–68].

### Olfaction- and taste-triggered immune response

Foreign substances in the URT can also be detected by two other systems, the extended olfactory and the trigeminal chemesthetic system. The former includes the olfactory epithelium and vomeronasal organ [69]. Stimulation thereof by different signals (food odors, sexual and social signals, as well as bacterial infection products like formyl peptides) was shown to cause behavioral responses in mouse experiments [70, 71].

The trigeminal chemesthetic system (including solitary chemosensory cells (SCCs)) [69] induces protective trigeminal nerve-mediated airway reflexes (coughing, sneezing, or decrease in breathing rate) and local inflammatory responses [72–74]. These SCCs make up to 1% of all cells in the ciliated epithelium of the sinonasal cavity [66, 75] and express two types of taste receptors, bitter and sweet [76, 77]. These receptors belong to the group of G-protein-coupled receptors (GPCRs) [78, 79].

With bitter receptors (e.g., T2R family), the sensory system of the SCCs is able to detect the presence of bacteria on nasal epithelial surfaces directly via bitter molecules that are released by pathogens [56, 73, 76] and may initiate immune responses (e.g., inflammation) even before bacteria achieve a pathogenic load and are able to form biofilms [38, 56, 80]. An example of a bitter, microbial-derived molecule is acyl-homoserine lactone (AHL). AHL is an important bacterial quorum-sensing molecule [36–38] that stimulates the bitter receptor T2R38 and leads to calcium-dependent nitric oxide (NO) production [56].

It should be noted that bitter and sweet signals affect innate immunity oppositely. Sugars, such as sucrose and glucose, inhibit bitter-induced calcium release. As a consequence, downstream, calcium-driven initiation of the innate immune system at the tissue level (such as release of antimicrobials from ciliated cells) is impaired [76, 80].

In patients suffering from prediabetes and diabetes, increased levels of glucose have been found in nasal secretions [81]. In addition, chronic rhinosinusitis patients reported higher intensity of the sweet tastes (sucrose) whereas their ability to taste bitter compounds was reduced compared to healthy controls, both leading to decreases in pathogen detection and defense, e.g., by reduced ciliary beating [38, 82, 83]. Furthermore, it is hypothesized that glucose levels in the airways rapidly deplete during a bacterial infection due to the bacterial load [82, 84].

## The upper respiratory tract microbiome changes with age and life-style

As we have seen, the landscape of the upper respiratory tract, with its different epithelial linings and conditions, provides numerous different (micro-)niches for microbial communities. Whereas the anterior naris (the passage between the skin and the nasal cavity) harbors commensals and opportunistic pathogens like *Staphylococcus aureus*, *S. epidermidis*, *Propionibacterium* (now: *Cutibacterium*) *acnes*, *Dolosigranulum pigrum*, *Finegoldia magna*, *Corynebacterium* spp., *Moraxella* spp., *Peptoniphilus* spp., and *Anaerococcus* spp. [85, 86], the microbial community structures in other locations in the nasal cavity and down the nasopharynx are distinct, especially in adults [9, 10] (see also Additional File 1). Even though the URT microbiome is largely individual, changes in inter-individual bacterial community profiles over different seasons (winter vs summer) and ages can still be observed [1, 86–89].

### The upper respiratory tract microbiome of infants

**Fig. 3:**
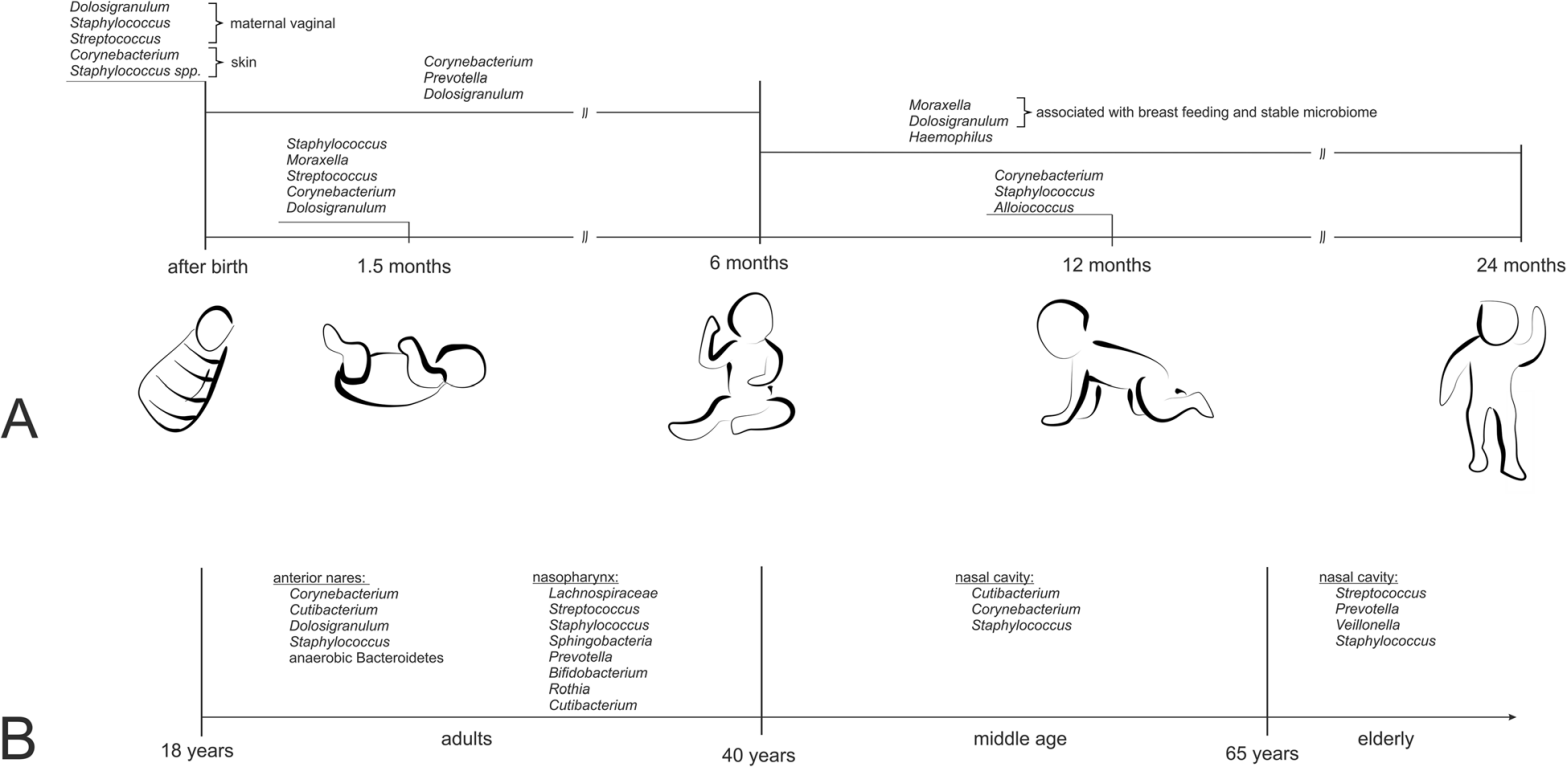
Nasal microbial composition during infancy and different age groups. **a** Directly after birth, infants’ URTs are colonized by maternal vaginal and skin bacteria. This initial URT microbiome changes during infant development. **b** The nasal microbiome is gradually reduced and microbial composition changes at different sampling sites. Bacterial genera given in the figure were found at or between the stated time points of life by molecular methods (16S rRNA sequencing with NGS). For references, see the text and Additional file 1

*Moraxella*, *Staphylococcus*, *Streptococcus*, *Haemophilus*, *Dolosigranulum*, and *Corynebacterium* are the six most common genera, of which one or two usually dominate the nares and nasopharyngeal microbiome of infants [11, 90, 91]. Right after birth, the initial nasopharyngeal bacterial assemblage takes place, and the infant’s nasopharyngeal microbiome resembles the maternal vaginal or skin microbiome [3, 92] (Fig. 3).

At 1.5 months of life this initial microbiome composition is maintained by breast feeding, which supports stable *Dolosigranulum*/*Corynebacterium* profiles. This is different to formula-fed infants, who show increased *S. aureus* signatures. The microbial profile of breast-fed infants seems to have a protective effect against respiratory infections [3, 93] (Fig. 3).

The nares and nasopharynx are dominated by *Staphylococcus*, *Moraxella*, *Streptococcus*, *Corynebacterium*, and/or *Dolosigranulum* signatures in 1.5-month-old infants [92]. Children with *Moraxella* spp.-dominated profiles were less likely to suffer from URTI, with the exception of *Moraxella catarrhalis*, which is found to be associated—together with *H. influenza* and *S. pneumoniae*—with wheezing in one-month-old infants. Nasopharyngeal *Streptococcus* was found to serve as a strong predictor for asthma in approximately 2-month-old children [27, 47, 92, 94]. After 1.5 months, *Haemophilus*-dominated clusters of co-occurrent microorganisms emerged, whereas *Staphylococcus*-dominated profiles disappeared and *Corynebacterium*/*Dolosigranulum* patterns were replaced by *Moraxella*/*Dolosigranulum*-dominated clusters in the infants’ URT [92] (Fig. 3).

All in all, observations of children in their first 2 years of life show that *Dolosigranulum* and *Moraxella* combined with *Corynebacterium* form a more stable microbiome compared to *Streptococcus* and *Haemophilus*-dominant profiles [26, 92]. The latter profiles (*H. influenza* and *S. pneumoniae*) were associated with respiratory viruses and an elevated risk of bronchiolitis in early life [30, 92, 95–97] (Additional file 1).

### The upper respiratory tract microbiome of adults

The URT microbiome of adults differs from that of infants, although the niche characteristics appear quite similar. In comparison, children’s nasal microbiomes are more dense (higher bacterial load) but less diverse [3, 8, 12, 47, 98]. The anterior nares of adults mainly harbor *Actinobacteria*, *Firmicutes*, and, in lower abundance, anaerobic *Bacteroidetes* [3, 31, 43, 98–100] (Fig. 3; Additional File 1).

Comparison of different nasal cavity sample sites showed that middle meatus (MM) and sphenoethmoidal recess (SR) are nearly identical with respect to microbial community composition, whereas anterior nares show a significantly reduced diversity of the microbial community. In addition, the anterior nares harbor a greater proportion of *Firmicutes* and *Actinobacteria* and less *Proteobacteria* compared to MM and SR [32].

The primary function of the nasal mucosa, namely the clearance of inhaled air, may explain the increased diversity of nasal mucosal samples [32]. At the phylum level, the adult nasopharynx microbiome resembles the microbiome of adult anterior nares, but the identified lower taxa are rather specific at the different locations [12] (Additional file 1).

### The upper respiratory tract microbiome of the elderly

The microbial communities of the anterior nares of adults (18–40 years) differ significantly from that of other URT sampling sites (nasopharynx, tongue, buccal mucosa, oropharynx), but these distinctive variations gradually reduce during ageing. The alterations in nasal microbiota communities start in middle-aged adults (40–65 years), whose nasal microbial communities are dominated by signatures of *Cutibacterium*, *Corynebacterium*, and *Staphylococcus*, whereas the nasal community of the elderly (> 65 years) shifts towards a more oropharyngeal population (Fig. 3) [9, 47]. These observed changes in bacterial community composition are probably a consequence of immune-senescence during the process of aging, which leads to an increase of pro-inflammatory markers and decreased ability of immune stress handling, leading to the opening of new environmental niches after the loss of species richness [9, 101] (Additional file 1).

### Smoking influences the nasal microbiome

Cigarette smoke exposure, whether active or passive, is associated with an elevated risk of not only cancer, periodontitis, and cardiovascular disease, but also chronic respiratory diseases (e.g., chronic obstructive pulmonary disease (COPD), asthma) and acute respiratory infections [47, 102].

Cigarette smoke has immediate contact with nasal surfaces, and thus directly impacts the microbiome by oxygen deprivation, antimicrobial activity, or other mechanisms [103, 104].

The toxic substances disrupt effective muco-ciliary clearance in the lower and upper respiratory tracts, impairing the immune responses against pathogens [105–109].

Cigarette smoke also enhances bacterial attachment to airway epithelial cells, for example, by inducing bacterial fimbrial protein FimA production, which promotes the formation of robust, reversible biofilms. This biofilm formation might support recalcitrant persistence of bacteria in the nasal cavity [87, 110–112].

Other studies suggested a direct alteration of bacterial infection and carriage pathways, as it has already been shown that *S. aureus* invasion and biofilm formation are elevated after cigarette exposure [47, 113, 114]. A similar effect was observed for pneumococcal biofilms [115, 116] (Additional file 1).

Several studies have shown that cigarette smoking depletes normal commensal airway microbiota and enriches potential pathogens (*H. influenzae*, *M. catarrhalis*, *Campylobacter* spp., *Streptococcus pneumoniae*, and *Streptococcus pyogenes)* [47, 87, 117]. In general, URT communities of smokers were found to be more diverse but less robust in composition over time compared to non-smokers [87] (Table 1; Additional file 1).

**Table 1:** Summary of significant URT microbiome changes due to active and passive cigarette smoking

Study	Population	Sample site	*Actinobacteria*	*Bacteroidetes*	*Firmicutes*	*Proteobacteria*
Charlson et al. 2010 [87]	Adult	Nasopharynx	↓*Actinomycetaceae* *↓Corynebacteriaceae* *↓Coriobacteriaceae* ↑*Eggerthella*	↓*Flexibacteriaceae* *↓Flavobacteriaceae* *↑Porphyromonadaceae*	↓*Leuconostocaceae* *↑Erysipelotrichaceae* *↑Aerococcaceae* *↑Eubacteriaceae* *↑Incertae Sedis XIII* *↑Peptostreptococcaceae* *↑Ruminococcaceae* ↑*Lachnospiraceae I.S.* spp. ↑*Anaerovorax* ↑*Dorea* ↑*Erysipelotrichaceae I.S.* ↑*Eubacterium* spp. ↑*Abiotrophia* spp.	↓*Rhodocyclaceae* *↓Rhodobacteraceae* *↓Enterobacteriaceae* *↓Alcaligenaceae* *↓Methylophilacea* ↓*Shigella* spp. ↑Pasteurellaceae ↑*Haemophilus* spp.
Brook and Gober 2005 [117]	Adult	Nasopharynx			↑*Streptococcus pneumoniae* ↑*Streptococcus pyogenes*	↑*H. influenzae* ↑*M. catarrhalis*
Greenberg et al. 2006 [118]	Infants	Nasopharynx			↑*Streptococcus pneumoniae*	
Sapkota et al. 2009 [119]	Not applicable	Cigarettes			***Bacillus*** ***Clostridium*** *Enterococcus* *Staphylococcus*	***Acinetobacter*** ***Burkholderia*** ***Klebsiella*** ***Pseudomonas aeruginosa*** ***Serratia*** *Campylobacter* *Proteus*

Several different microbial signatures of the phyla Actinobacteria, Bacteroidetes, Firmicutes and Proteobacteria have been found to be altered in humans exposed to cigarette smoke. Arrows indicate an increase (↑) or decrease (↓) in relative abundance in smokers compared to non-smoking subjects. Signatures of ***Bold*** microbial genera were found to be present in more than 90% of all cigarette samples (Additional file 1)

The likelihood of carrying Gram-positive anaerobic lineages (*Eggerthella*, *Erysipelotrichaceae I.S.*, *Dorea*, *Anaerovorax*, and *Eubacterium* spp.) is increased in the nasopharynx of smokers, including pathogens associated with URT infections and endocarditis (e.g., *Abiotrophia* spp.) [87] (Table 1; Additional file 1). In contrast, the upper respiratory tract of non-smokers harbors particularly *Peptostreptococcus* spp., α-haemolytic streptococci, and *Prevotella* spp., which seem to correlate negatively with pathogen presence [47, 117].

Interestingly, after 1 year (12 to 15 months) without smoking, the microbiome composition seems to recover and resembles microbial patterns of never-smokers, accompanied by a decrease of the proportion of opportunistic pathogens [87, 111, 120] (Table 1).

Smoking is not only harmful for adults, but also for infants when they are exposed to passive smoking. In general, *S. pneumoniae* was found to be elevated in infants with smoking parents [118]. Two-year-old children of smoking parents also have an increased risk of suffering from otitis media, meningococcal meningitis, and lower respiratory tract infections [111, 121, 122] (Additional file 1).

Notably, cigarettes themselves could be the source of these opportunistic pathogens. Sapkota et al. studied the bacterial metagenomes of commercially available cigarettes and discovered signatures of, e.g., *Acinetobacter*, *Burkholderia*, *Clostridium*, *Klebsiella*, *Pseudomonas aeruginosa*, and *Serratia* [119] (Table 1; Additional file 1).

## Microbial competition in the URT

**Fig. 4:**
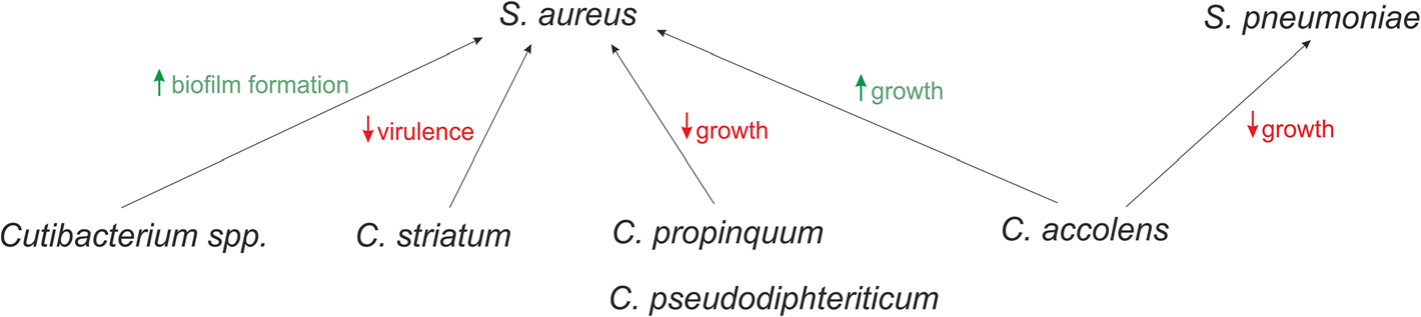
Influence of nasal microbial community members on each other. Different *Corynebacterium* spp. are able to inhibit as well as promote growth of staphylococci and *S. pneumoniae* in vitro, whereas other species led to increased biofilm formation and reduced virulence. For references, see the text

Most microbes associated with the human host interact positively with the host and each other. This collaboration is mostly based on syntrophic (i.e., co-feeding) networks [123]. However, if certain resources are restricted, or niches overlap, competitive interactions can occur between commensals (Fig. 4) and with opportunistic pathogens and the host. These interactions can involve direct and indirect attack of competitors.

For instance, microbes, colonizing the upper airways, have to cope with a scarcity of freely available glucose and iron [124–127]. To overcome these limitations, microbes can either scavenge iron from human cells [124] or release iron-chelating molecules (siderophores) that bind ferric iron from the adjacent environment [128].

Understanding the mechanisms of direct (e.g., secretion of antimicrobial peptides) and indirect microbial competition actions within the URT may illuminate new approaches for the development of new antimicrobial therapies for various diseases, for example, those caused by *Staphylococcus aureus* or *Streptococcus pneumoniae* [32, 129–132].

Although studies on microbe–microbe interaction also focus on other abundant genera of the human upper airways [1, 86, 132–138], knowledge on microbial competition for potential treatment of *S. aureus* infections is particularly important. This opportunistic pathogen is an asymptomatic colonizer of human skin and nose but it is also able to cause chronic and indolent to acute and aggressive infections in cases of excessive overgrowth [139–141].

One potentially applicable agent for a putative therapy is secreted by S. *lugdunensis*, namely lugdunin (a thiazolidine-containing cyclic peptide), which inhibits the growth of *S. aureus* in vitro [142]. Another candidate is the antimicrobial peptide nukacin IVK45, produced by *S. epidermidis IVK45* under in vitro oxidative stress and iron limitation [130, 143]. Species- or even strain-specific inhibition or promotion of staphylococci has also been observed for *Corynebacteria* [32, 129, 130]. Whereas some *C. pseudodiphteriticum* were able to inhibit the growth of *S. aureus*, co-cultivation with *C. accolens* led to supported and enhanced growth of both strains, indicating a possible cooperative interaction [32].

*Corynebacterium* species, or even cell-free conditioned medium thereof, were found to shift *S. aureus* towards a more commensal state and attenuation of virulence by downregulation of components involved in colonization and virulence, such as the agr operon or genes involved in hemolytic activity [129, 144, 145].

In contrast, methionine synthesis and iron acquisition were found to be upregulated in *S. aureus* when co-cultured with *C. striatum*. Based on this observation, Ramsey et al. envisage a competitive situation for methionine and iron in vitro [129]. It should be noted that coagulase-negative staphylococci are more sensitive to these types of nutrient competitions, as they produce lower levels of siderophores; however, resulting growth inhibition has been abolished by providing iron supplementation [124, 130, 146].

Besides Corynebacterium, *Cutibacterium* spp. (and its cell-free conditioned medium) is also able to affect growth of *S. aureus*. Coproporphyrin III (CIII), the most abundant porphyrin secreted by *Cutibacterium* ssp., induces *S. aureus* aggregation and biofilm formation in culture. Therefore, it also might promote biofilm formation with other members of the nostril’s microbial community [132, 147, 148].

*S. pneumoniae*, a common inducer of URT diseases such as pneumonia, sinusitis, or otitis media [131, 149], can be inhibited by *C. accolens* through the production of free fatty acids (FFAs) from the host’s triacylglycerols (TAG), causing an increase in the expression of antibacterial human β-defensin-2 [131, 150, 151].

## Non-bacterial microorganisms in the human nose

Besides bacterial and viral components, the nasal cavity contains a unique, highly diverse archaeal community. Archaea are microorganisms that are, due to their different biology, distinctive from bacteria. They are also relevant components of the human microbiome inhabiting the gastrointestinal tract, oral cavity, skin, and other areas [152]. The archaeal community of the nasal cavity resembles that of the archaeomes of skin and the intestinal tract in being dominated by skin-associated Thaumarchaeota (*Nitrososphaera*) and also methanogenic Euryarchaeota (*Methanosphaera*, *Methanobrevibacter*) which are characteristic of the archaeal communities in the gastrointestinal tract [13]. Notably, the nasal cavity was found to represent an archaeal hot-spot amongst other body sites, with a high archaeal 16S rRNA gene content [153]. The importance of archaea in the nasal cavity was supported by a recent correlation of methano-archaeal presence in refractory sinusitis [154].

Severe knowledge gaps also exist with respect to the mycobiome and virome of the upper respiratory tract; as these fields are not part of this review, we refer readers to some recent reviews on these topics [96, 155–158] (Additional file 1).

## Correlations between the upper respiratory tract microbiome and disease

The anterior nares are an open environment and in contact with several thousands of liters of inhaled air every day [159]. Therefore, besides the gastrointestinal tract, the nasal cavity has been suggested to represent the main entry port for pathogens, pollutants, and pollen, potentially causing imbalances in the nasal microbial community composition [89, 160, 161]. Microbiome dysbiosis is considered an important biomarker for human disease such as chronic rhinosinusitis [6, 162].

### URT microbiome diversity and specific health-associated bacteria are reduced in chronic rhinosinusitis

Chronic rhinosinusitis (CRS) is a common chronic and detrimental inflammatory disorder of the human paranasal sinuses. It lasts for more than 12 weeks and affects up to 16% of the population [15, 163, 164]. Although CRS is suggested to be an inflammatory disease rather than an infectious one, bacterial contributions to the initiation and progression of inflammation are important to consider [165–167].

Previous studies suggested a polymicrobial process behind CRS [168]. A decrease in microbial diversity, richness and evenness, which are frequent features in other chronic inflammatory diseases as well, has been observed in CRS patients in several studies [15, 20, 47, 169–171]. This decline may occur due to an elevated presence of anaerobic bacteria growing in biofilms [172, 173]. Notably, the overall bacterial burden and phylum level abundance were found to remain constant, whereas the relative abundance of specific bacterial genera is altered in CRS patients [171, 174]. Hoggard et al. reported a depletion of signatures of *Anaerococcus*, *Corynebacterium*, *Finegoldia*, *Peptoniphilus*, *Propionibacterium*, and *Staphylococcus* in CRS patients—all previously identified as typical health-associated URT bacteria [162, 170]. This shift away from a healthy microbial community may lead to an increase of both inflammatory response (Toll-like receptor responses) and clinical severity [20, 175] (Table 2; Additional file 1).

**Table 2:** The nasal microbiome of chronic rhinosinusitis patients

Study	Population	Sample site	*Actinobacteria*	*Bacteroidetes*	*Firmicutes*	*Proteobacteria*
Lal et al. 2017 [29]	Adults with nasal polyps	Middle meatus	*Streptococcus* *Haemophilus* *Fusobacterium*			
	Adults without nasal polyps	Middle meatus	*Corynebacterium*		*Staphylococcus Alloiococcus*	
Copeland et al. 2018 [6]	Adults	Middle meatus	*↓Corynebacterium*	*↑Porphyromonas* *↑Prevotella*	*↑Anaerococcus* *↑Lactobacillus* *↑Finegoldia* *↑Peptoniphilus* *↑Dialister* *↑Parvimonas* *↓Staphylococcus* *↓Dolosigranulum*	
Hoggard et al. 2018 [20]	Adults	Middle meatus	*↓Corynebacterium* *↓Propionibacterium*		*↓Anaerococcus* *↓Finegoldia* *↓Peptoniphilus* *↓Staphylococcus*	
Aurora et al. 2013 [176]	Adults	Middle meatus	*↑Corynebacterium* *↑Curtobacteria*		*↑Staphylococcus*	*↑Pseudomonas*
Cope et al. 2017 [168]	Adults	Sinus	*Corynebacteriaceae*		*Staphylococcaceae* *Streptococcaceae*	*Pseudomonadaceae*

Arrows indicate an increase (↑) or decrease (↓) in relative abundance in CRS patients compared to healthy subjects. Relative abundance was analyzed by 16S rRNA sequencing

A study on sinus microbiomes reported that most sinuses of CRS patients are dominated by signatures of *Corynebacteriaceae*, *Pseudomonadaceae*, *Staphylococcaceae*, or *Streptococcaceae.* These bacterial families were found to co-occur with a unique set of bacterial taxa with lower abundance [168] (Table 2). Other studies showed an overgrowth of *Corynebacterium tuberculostearicum* and *Staphylococcus* enrichment in sinuses [15, 169], as well as *Corynebacterium*, *Curtobacteria*, *Pseudomonas*, *Staphylococcus*, or *H. influenza* enrichment in the middle meatus [176, 177] (Table 2).

In the middle meatus, Copeland et al. found a negative correlation of the CRS disease state and six OTUs (operational taxonomic units) affiliated to genera *Staphylococcus*, *Corynebacterium*, and *Dolosigranulum*. *Corynebacterium* OTU410908 was the only signature to correlate negatively with the SNOT-22 (Sinonasal Outcome Test) score, which states disease severity [6] (Table 2).

Generally, anaerobic genera (*Anaerococcus*, *Lactobacillus*, *Finegoldia*, and *Peptoniphilus*) were found to be more present in CRS patients’ compared to healthy subjects’ middle meatuses [6] (Table 2; Additional file 1).

Traditionally, CRS is categorized in two subtypes: CRS with the absence (CRPsNP) or presence (CRPwNP) of nasal polyps (fleshy swellings arising due to inflammation) [6, 15, 163]. Notably, in CRSwNP patients, comorbidities such as aspirin intolerance and asthma are likely to occur [177]. Comparing the inferior and middle meatus microbiome of these different phenotypes reveals that CRSwNP samples were enriched by signatures of *Alloiococcus*, *Staphylococcus*, and *Corynebacterium* spp., whilst CRSsNP patients were enriched mainly by anaerobes, such as *Haemophilus*, *Streptococcus*, and *Fusobacteria* spp., and showed depletion of *Rothia*, *Alloiococcus*, *Corynebacterium*, and *Finegoldia*. Usually, the sinus cavities are not anaerobic; therefore, this enrichment of anaerobes in CRPsNP subjects is probably a result of disease progression and pathology [178]. *Fusobacteria*, for example, are associated with suppuration, which can cause anaerobic conditions in the paranasal cavities [29, 176] (Table 2; Additional file 1). Additionally, the severity of inflammation was positively correlated with the phylum Bacteroidetes (e.g., *Prevotella*) and the phylum Proteobacteria (*Pseudomonas*) in CRS [179].

Another interesting aspect is that CRS patients have an altered response to taste molecules. They are less sensitive to bitter while being more sensitive to sweet molecules [83]. As described above, bitter receptors in the nose play an important role in bacterial detection and defense. As a result of these alterations CRS patients have less stimulation of ciliary beating in the URT and show altered NO levels [38, 180]. Notably, It has already been shown that the functional capability of these taste receptors in the URT correlates with severity of CRS [80, 83, 181, 182].

Nasal washes, corticosteroids, and sinus surgery are the most common treatments for CRS and may significantly influence the URT microbiome. The therapy options and their effects are discussed later in this review [21–24].

### Nasal microbiome composition may be linked to neurological diseases

Some reports indicate a potential involvement of the (nasal) microbiome in Parkinson’s disease (PD), Alzheimer’s disease (AD), and multiple sclerosis (MS) [183]. In particular in PD and AD, the first symptoms are olfactory dysfunction (see below), and a link with the nasal microbiome of the olfactory area has been hypothesized as microorganisms contribute to normal development of the olfactory epithelium [184]. Since the nasal microbiome in AD and MS have not been studied in detail yet, we herein concentrate on PD as an example. PD is a neurodegenerative disease that is characterized by clumping of the protein α-synuclein in neuronal cells. In the dopaminergic substantia nigra of the central nervous system (CNS), these aggregates, also called Lewy bodies, lead to neuronal loss [185, 186]. α-Synuclein pathology was found to affect olfactory bulb function [160, 185, 186], and more than 90% of PD patients suffer from decreased olfactory function or hyposmia, even before motor symptoms occur [187].

Some studies suggested that a failure in innate immune system priming by nasopharyngeal microbiota could lead to an inflammatory response to α-synuclein, oxidative stress, cross-seeded misfolding, and thus development of neurodegenerative diseases [188–191]. Therefore, the studies hypothesized that the microbial community contributes to the initiation of PD [187, 192, 193].

No significant differences in alpha and beta diversity between the nasal microbiome of PD patients and healthy participants had until now been observed [192]. However, Pereira et al. showed that two taxa were less abundant in PD patients compared to healthy controls, namely signatures of the family *Flavobacteriaceae* and the genus *Marmoricola* [192] (Additional file 1).

Other studies hypothesize that a currently unknown, transmissible infectious agent enters the brain through the gastrointestinal tract and/or the nasal cavity and initiates the pathological process in the CNS [160, 193].

However, this research is at an early stage and the importance of the microbial community in initiation of PD requires further investigation.

#### The respiratory tract microbiome of cystic fibrosis patients follows clear patterns and might be established already early in life

Cystic fibrosis (CF) is a hereditary life-limiting disease that is caused by mutations in the gene of the cystic fibrosis transmembrane conductance regulator (CFTR). It can affect diverse organs but in most cases results in chronic lung disease [117, 120], characterized by a defect in mucociliary clearance and mucopurulent secretions [194–197]. The lungs of CF patients are colonized with so called “typical CF pathogens” consisting of bacterial genera *Rothia*, *Prevotella*, *Streptococcus*, *Actinomyces*, and *Veillonella* [195, 198, 199]. In addition to this so-called CF core microbiota, other CF-associated pathogens like *Pseudomonas aeruginosa*, *Haemophilus influenza*, *Burkholderia cepacia complex*, and *Staphylococcus aureus* can lead to chronic lung infection in CF [16, 194, 195]. The microorganisms originating from the environment probably spread via inhalation or micro-aspiration from the upper respiratory tract (URT) into the lungs [194, 200]. Several studies also demonstrate that the nasal cavity and the nasopharynx act as a reservoir for further colonization of these potential respiratory pathogens (PRPs), before they spread in the lower airways [26, 201, 202] (Additional file 1).

In CF infants, the nasal microbiome shows significant differences when compared to healthy controls. For instance, the relative abundance of *Corynebacteriaceae* and *Pastorellaceae* signatures was found to be reduced in the nasal microbiome of CF infants, whereas the relative abundance of *Staphylococcaceae* was increased. In nasopharyngeal samples, *S. mitis*, *Corynebacterium accolens*, and *S. aureus* as well as Gram-negative bacteria were more abundant in CF children [90]. This increased abundance of *S. aureus* in CF infants in early life is probably caused by a defect of the early innate immune system; moreover, due to accumulation of mucus, microaerobic conditions prevail in the airways of CF patients, which could lead to a better survival of *S. aureus* [26, 203, 204]. The URT microbiome of CF children adult CF patients is very similar, indicating establishment of this abnormal microbiome early in life [194] (Additional file 1).

## Nasal microbiome in olfactory function and dysfunction

The functional area of human olfaction in the nose is the olfactory mucosa, which is located at the ceiling of the nasal cavity, is 8 to 10 mm long, and extends from the septum to the middle and superior turbinate. This olfactory area is characterized by a high abundance of bipolar neurons from the olfactory nerve and the presence of lactoferrin, IgA, IgM, and lysozyme, which prevent pathogens from intracranial entry through the cribriform plate [205].

The olfactory receptor cells in the olfactory mucosa pass through the cribriform plate into the olfactory bulb of the CNS. These cells are able to recognize different odor molecules, but also secondary metabolites of bacteria [33, 206]. In general, microbes are known to be able to interact with human body tissues via secondary metabolites, including short-chain fatty acids and other, hormone-like molecules [207–209].

Most cases of olfactory loss occur secondary to inflammation (caused, for example, by viral infections or chronic rhinosinusitis), traumatic brain injuries, ageing, or neurodegenerative diseases (e.g., PD and Alzheimer’s disease) [210, 211]. In addition, as the physiology of the olfactory epithelium can be modulated by the microbiome, an influence of the microbial composition on olfactory function and dysfunction has been suggested [43, 184].

In healthy, normosmic volunteers Koskinen et al. identified four archaeal and 23 bacterial phyla in the microbiome of the olfactory area, the latter with *Actinobacteria*, *Firmicutes*, *Proteobacteria*, and *Bacteroidetes* predominating. On the genus level, signatures of *Corynebacterium*, *Staphylococcus*, and *Dolosigranulum* were shown to be most abundant [43]. *Corynebacterium* and *Staphylococcus* are typical human skin bacteria, frequently found in the nasal cavity [1, 134, 138, 212, 213]. *Dolosigranulum* has been observed to be a health-associated commensal inhabitant [139], but *Dolosigranulum pigrum*, an opportunistic pathogen, can, under certain conditions, also cause infections [214, 215] (Additional file 1).

Besides the healthy, normosmic participants, subjects with different olfactory performance were also studied [43]. Olfactory performance can be assessed by three different metrics: odor threshold (T; lowest concentration of odor compound perceivable), odor discrimination (D; discrimination of different odors), and odor identification (I; identification/naming of a certain odor). Based on these scores an overall TDI score is calculated. This TDI score categorizes subjects as normosmics (with normal olfactory performance), hyposmics (with decreased olfactory function), and anosmics (complete loss of olfactory function) [216, 217].

It is thought that an impacted nasal airflow influences the URT microbiome indirectly by changing local parameters (such as humidity, temperature, oxygenation). Such impacted airflow can occur due to rhinosinusitis, allergic rhinitis, head trauma, nasal surgery or congenital causes [33, 218–220] and might also contribute to the decrease in olfactory function by affecting the microbial community structure.

Indeed, Koskinen et al. observed that the microbiome of hyposmic subjects differed significantly in community composition and diversity compared to normosmics [43]. Odor threshold hyposmics (people with poor T score) showed a higher microbial diversity at the olfactory area, for example, signatures of the genus *Campylobacter* were found to correlate negatively with this condition, whereas *Proteobacteria*, *Actinobacteria*, *Firmicutes*, and *Bacteroidetes* were associated with poor odor identification. Furthermore, butyrate-producing bacteria like *Faecalibacterium* correlated negatively with odor threshold and discrimination, *Enterobacteriaceae* correlated negatively with odor threshold and identification, and *Porphyromonas* and unclassified *Lachnospiraceae* correlated negatively with overall olfactory performance (T, D, I) [43]. Whereas *Porphyromonas* is a typical representative of the human oral microbiome, *Faecalibacterium*, *Enterobacteriaceae*, and *Lachnospiraceae* are gut microorganisms, capable of producing butyrate. As butyrate has a very strong and unpleasant odor, and the production is out of place in the nasal area, it was suggested that it may have an impact on olfactory performance [43, 167, 221] (Additional file 1).

Analyzing the microbial composition and abundance with the goal of providing therapy options (e.g., through probiotics) could be one possible way to improve life quality for the 20% of the general population suffering from olfactory dysfunction.

## Therapies change the URT microbiome composition and diversity

Intranasal corticosteroids (INS), saline rinses, antihistamines, and antibiotics are the current medical therapies of choice for inflammatory disorders of the upper respiratory tract [21, 24]. In contrast to anti-inflammatory substances that act through immunomodulatory mechanisms, antibiotics and some INS have antimicrobial properties and thus impact the microbial community directly [24, 222].

### Antibiotics and other intranasal medication

Antibiotics and other medication with antimicrobial properties are usually used to treat severe bacterial infections. However, in some cases they are applied prophylactically, for example, before sinus surgery to diminish the bacterial load in the nasal cavity [24].

Application of antibiotics has been shown to influence microbial community composition significantly by reducing the microbial diversity not only in the gut, but also in the upper respiratory tract of infants and adults. The shift in the URT microbial profile results in an increased abundance of Gram-negative bacteria (*Burkholderia*, *Comamonadaceae*, *Bradyrhizobiaceae*, and *Enterobacteriaceae*) as well as *Moraxella*, *Haemophilus*, *Staphylococcus*, and *Streptococcus* [25–27]. Under normal circumstances, these bacteria are unable to compete in this niche, but due to tolerance to several antibiotics (e.g., *H. influenza* and *Chlamydia pneumoniae*: resistance towards β-lactam antibiotics; *S. pneumoniae*: resistance towards aminoglycosides, fluroquinolones, and β-lactam) they are able to expand during antibiotic treatment and become pathogenic [223, 224]. In contrast, abundances of known commensals such as *Dolosigranulum* and *Corynebacterium*, which normally are highly abundant in the human nose and associated with decreased URT infection risk and microbiota stability, are reduced by the treatment. These shifts in the anterior nares microbiome lasted throughout treatment and even posttreatment period (at least 2 weeks after treatment) [24, 93].

Topical antibiotic therapy with, e.g., mupirocin is used as standard preoperative therapy for non-allergic rhinitis (i.e., chronic rhinosinusitis). It has been shown that antibiotic treatment with muropirocin was able to decolonize *S. aureus* preoperatively, decreasing *S. aureus* site infections in surgery [24, 225, 226].

INSs like mometasone furoate monohydrate, which has anti-inflammatory properties, are common first line therapies for allergic rhinitis (AR) [21, 24]. INSs affect the composition and biodiversity of the nasal microbiome: like antibiotics, this medication suppresses several taxa (*Moraxella* spp., streptococci) and may promote the dominance of other taxa such as staphylococci [24, 225, 226].

### Alterations in nasal structure due to sinus surgery influence the microbial community in the nasal cavity

Endoscopic sinus surgery (ESS) is an invasive treatment mainly used for polyposis and refractory sinusitis [22]. It enlarges the size of sinus ostia, improves mucociliary clearance, and facilitates access for topical therapies [218]. This intervention changes the physical sinus structure and may influence paranasal physiology by reducing the temperature and humidity in the nasal cavity. This drier and cooler post-operative ecosystem might have an effect on microbial composition and metabolism [218, 227].

Overall, the post-operative outcome of the surgery is positive, and only a subset of the patients does not recover [28, 228]. This subset suffers from a recolonization by pathogens despite antibiotic treatment after surgery [229–231]. It is suggested that the repopulation has its origins in paranasal sinus biofilms or in the nasopharynx, as these areas are better protected from antibiotics [164, 229, 232, 233]. It has also been reported that CRP patients who suffer from inflammation after the surgery have higher numbers of SCCs in the URT inflamed tissue [66]. Furthermore, patients with the non-functional genetic variation of the bitter receptor T2R38 are more likely to need surgery and develop bacterial infections [82, 83].

Notably, Hauser et al. found that the bacterial load of the ethmoid is lower at the time of surgery and 6 weeks after surgery than in the postoperative period (2 weeks after surgery). The authors suggested that a broad disruption of immune function and the mucociliary system due to the surgical intervention is responsible for this altered bacterial burden [229].

In an independent study, Jain et al. [218] reported an increase in the number of bacterial signatures, but no change in overall microbial profile 4 months after surgery compared to pre-operative microbial profiles. However, the relative abundance of *Staphylococcus* signatures increased whereas *Streptococcus* and *Corynebacterium* decreased; most changes were observed in extremely low-abundance taxa (e.g., *Peptoniphilus*, *Finegoldia*, *Faecalibacterium*, *Campylobacter*) [218].

Other studies reported similarities between the bacterial community of the ethmoid and sinuses after surgery and those of the anterior nasal cavity and pretreatment sinuses, and also the presence of bacteria from extra nasal sources, suggesting that all these sites serve as likely sources for recolonization [164, 229, 233, 234].

### Nasal rinse might be a microbiome-friendly alternative to aggressive therapy options for URT diseases/problems

Nasal rinse has its origins in Ayurveda, an ancient, traditional system of Indian healthcare [235]. Today, nasal rinse is not only used to treat upper respiratory tract problems, as URTIs, CRS, or AR, but also as prevention of those diseases. Nasal irrigation is thought to clean the nasal mucosa from inflammatory mediators like leukotrienes and prostaglandins, antigens, and other pollutants [23, 236, 237]. The most common rinsing solutions are isotonic saline (0.9%) or hypertonic saline (1.5–3%), pH varying from 4.5 to 7, but distilled, tap, and well-water is also used [23, 238].

The potential microbial contamination of irrigation water and devices has been of concern, as it might contain *S. aureus* and *Pseudomonas* spp. which cause the majority of postoperative infections [234, 238, 239]. However, these low abundance contaminations showed only little impact on microbial composition in the human sinonasal cavity [240]. Nevertheless, distilled water is recommended, as tap water and well-water can also lead to mycobacterial infections and amebic brain abscesses [238, 241, 242].

The high frequency of positive results of nasal irrigation in several studies indicates that nasal rinsing is an effective, inexpensive, and simple method to treat sinonasal disorders alone or in association with other therapies to reduce medicine consumption.

### Probiotics might be a non-invasive disease prevention and therapy option

In many cases of asthma and CRS, microbial dysbiosis is manifested by the expansion of pathogens and the loss of beneficial microorganisms [243, 244]. Living beneficial bacteria (probiotics) administered in adequate amounts can provide health benefits to the host [19, 245, 246]. Probiotic species may act as pioneers after disruption due to antibiotics, or have a larger beneficial effect on the community by acting as keystone species [247]. Additionally, probiotic strains may even be able to improve the epithelial barrier (by modulation of signaling pathways [248, 249]) or to interact positively with the host innate immune system [245, 246, 250, 251]. Probiotic microbes can interact with other microbes of the human microbiome by production of antimicrobials, competitive colonization, and inhibition of pathogen growth (e.g., by changing the pH in the niche) [247, 252, 253]. Probiotic bacteria can have various immunomodulatory functions, including T helper cell 1 (Th1)/T helper cell 2 (Th2) immune balance restoration, stimulation of regulatory T cells (Treg), the regulation of regulatory cytokines [254–257], and also the modulation of allergen-specific T- and B-cell responses and mucosal IgA levels [258].

Immune cells, microbial metabolites, and cytokines released due to oral probiotic supplementation reach the airways through translocation into the blood and systemic circulation, whereas probiotics applied via nasal sprays affect the local immune response and the sinonasal microbiome [259–263]. For example, *Lactobacillus rhamnosus* leads to an increase in Th1 and decrease in Th2 levels in mice [264, 265], and treatment of acute sinusitis in children with *Enterococcus faecalis* has already been shown to reduce frequency and duration of sinusitis [246, 266].

The next logical step would be the application of probiotics nasally, although a potential risk of inflammation in the lower airways due to aspiration into the lung might exist [246, 267]. However, Martensson et al. were able to show, although no significant effects on CRS disease progression were observed, that nasal application of 13 honeybee lactic acid bacteria (various *Bifidobacteria* and lactobacilli of the honey stomach of *Apis mellifera*) was well tolerated by patients. This probiotic was able to restore commensal microbiomes and to prevent infections through antibacterial activity. Furthermore, no side effects could be observed [246, 268–272].

## Knowledge gaps, conclusion, and outlook

Research on the microbiome of the URT has already revealed insights into its dynamic niche-specific composition, interactions between microbes and the host’s immune, olfactory, and chemosensory systems, and alterations that are associated with age, lifestyle and disease. This research is, however, still in its infancy. The majority of current knowledge about the URT microbiome is based on cultivation assays, targeting only a fraction of the microbial community, or next generation sequencing of segments of the bacterial 16S rRNA gene amplified from uncultured samples. These short reads provide basic information about the diversity and taxonomic composition of bacterial communities. However, more accurate species or strain level community profiling can now be achieved using, for example, long-read technologies for sequencing the entire 16S rRNA gene, such as Oxford Nanopore [273] or Pacific Bioscience (PacBio) technology [274], which has already been successfully applied to analysing the healthy sinonasal microbiome [275]. Shot-gun metagenomics is another approach that is increasingly used in microbiome research, offering insights into microbial genomes and functions, and the possibility to assemble draft genomes of uncultured human health or disease associated microbes. Untargeted shot-gun metagenomics could also give unbiased insights into the archaeome, mycobiome and virome of the URT, although due to the low abundance of many of these components, targeted approaches could be more effective in capturing their full diversity.

Determining whether the detected changes or dysbioses in the URT microbiome associated with disease are markers or drivers presents a major challenge. There has already been some progress towards identifying biomarkers that could be used for early diagnosis of URTIs, such as *Microbacterium spp., Streptococcus spp*. or *Faecalibacterium spp.,* whereas identifying targets for microbiome-based therapies remains more difficult. The ability to sample from disease-relevant sites within the URT is helpful in this regard, as it enables the identification of microbial candidate disease drivers whose abundance is positively correlated with both the site and incidence of disease, while negative correlations reported from the disease site are similarly more likely to be relevant, pointing to a possible protective role that might be harnessed in probiotic therapy. It will therefore be important to address the methodological challenges of sampling from less accessible URT sites, and to continue to develop appropriate sampling tools to minimise contamination from neighbouring sites. Further investigation of the co-operative and competitive interactions of microbes and host may also be helpful in guiding rational choices in the pursuit of causal connections and therapeutic goals. However, establishing causality and demonstrating the efficacy of proposed treatments requires other approaches, such as animal models and clinical trials.

Physicians and patients have high expectations of microbiome-driven therapies, yet most available knowledge stemming from basic research or clinical trials is far from impacting, or being implemented in, medical treatment. The results we have surveyed in this review suggest there are good reasons to remain optimistic about therapeutic solutions emerging from URT microbiome research, especially as newly available methodologies are deployed and current knowledge gaps are filled.

## Supplementary information

**Additional file 1.** Summary of the URT microbiome during the process of aging and in health and disease (collated information from selected studies).

## Data Availability

Not applicable.

## Abbreviations

URT: Upper respiratory tract
URTI: URT infections
CRS: Chronic rhinosinusitis
AN: Anterior nares
MM: Middle meatus
OR: Olfactory area
SR: Sphenoethmoidal recess
COPD: Chronic obstructive pulmonary disease
OTU: Operational taxonomic unit
CRPsNP: CRS with the absence of nasal polyps
CRPwNP: CRS with the presence of nasal polyps
PD: Parkinson's disease
CNS: Central nervous system
CF: Cystic fibrosis
CFTR: Cystic fibrosis transmembrane conductance regulator
PRPs: Potential respiratory pathogens
Ig: Immunoglobulin
T: Odor threshold
D: Odor discrimination
I: Odor identification
INS: Intranasal corticosteroids
ESS: Endoscopic sinus surgery
AR: Allergic rhinitis
GIT: Gastrointestinal tract
NGS: Next generation sequencing
